# Predictive Values of Programmed Cell Death-Ligand 1 Expression for Prognosis, Clinicopathological Factors, and Response to Programmed Cell Death-1/Programmed Cell Death-Ligand 1 Inhibitors in Patients With Gynecological Cancers: A Meta-Analysis

**DOI:** 10.3389/fonc.2020.572203

**Published:** 2021-02-01

**Authors:** Chen Zhang, Qing Yang

**Affiliations:** Department of Obstetrics and Gynecology, Shengjing Hospital of China Medical University, Liaoning, China

**Keywords:** gynecological cancers, programmed death ligand 1, prognosis, immunotherapy, clinicopathological features

## Abstract

**Background:**

The prognostic value of programmed cell death-ligand 1 (PD-L1) in gynecological cancers has been explored previously, but the conclusion remains controversial due to limited evidence. This study aimed to conduct an updated meta-analysis to re-investigate the predictive significance of PD-L1 expression.

**Methods:**

PubMed, EMBASE and Cochrane Library databases were searched. The associations between PD-L1 expression status and prognosis [overall survival (OS), progression-free survival (PFS), recurrence-free survival (RFS), cancer-specific survival (CSS) or disease-free survival (DFS)], clinical parameters [FIGO stage, lymph node metastasis (LNM), tumor size, infiltration depth, lymphovascular space invasion (LVSI) or grade] and response to anti-PD-1/PD-L1 treatment [objective response rate (ORR)] were analyzed by hazard ratios (HR) or relative risks (RR).

**Results:**

Fifty-five studies were enrolled. Overall, high PD-L1 expression was not significantly associated with OS, PFS, RFS, CSS and DFS of gynecological cancers. However, subgroup analysis of studies with reported HR (HR = 1.27) and a cut-off value of 5% (HR = 2.10) suggested that high PD-L1 expression was correlated with a shorter OS of gynecological cancer patients. Further sub-subgroup analysis revealed that high PD-L1 expressed on tumor-infiltrating immune cells (TICs) predicted a favorable OS for ovarian (HR = 0.72), but a poor OS for cervical cancer (HR = 3.44). PD-L1 overexpression was also correlated with a lower OS rate in non-Asian endometrial cancer (HR = 1.60). High level of PD-L1 was only clinically correlated with a shorter PFS in Asian endometrial cancer (HR = 1.59). Furthermore, PD-L1-positivity was correlated with LNM (for overall, ovarian and endometrial cancer expressed on tumor cells), advanced FIGO stage (for overall, ovarian cancer expressed on tumor cells, endometrial cancer expressed on tumor cells and TICs), LVSI (for overall and endometrial cancer expressed on tumor cells and TICs), and increasing infiltration depth/high grade (only for endometrial cancer expressed on TICs). Patients with PD-L1-positivity may obtain more benefit from anti-PD-1/PD-L1 treatment than the negative group, showing a higher ORR (RR = 1.98), longer OS (HR = 0.34) and PFS (HR = 0.61).

**Conclusion:**

Our findings suggest high PD-L1 expression may be a suitable biomarker for predicting the clinical outcomes in patients with gynecological cancers.

## Background

Gynecological cancers have been a significant global health burden for women (1, 2). According to the statistics by the American Cancer Society in 2020, uterine corpus endometrial cancer accounts for approximately 65,620 new cases and 12,590 deaths, followed by ovarian cancer (21,750 new cases and 13,940 deaths) and cervical cancer (13,800 new cases and 4,290 deaths) (3). Although several therapeutic options (i.e. surgery, chemoradiotherapy and immunotherapy) have been recommended recently, some patients exhibit a poor response to these management strategies and experience relapses or metastases, ultimately dying from their diseases (4). Therefore, predictive biomarkers may be urgently necessary to early stratify these patients at a high risk of poor responses and unfavorable outcomes and then guide more individualized treatment regimens to further improve overall survival (OS).

Recently, accumulating evidence has revealed that immune escape represents a crucial hallmark for malignant transformation and tumor progression (5, 6). The programmed death-ligand 1 (PD-L1, also called B7-H1 or CD274)/programmed cell death-1 (PD-1) axis is a major immune checkpoint pathway (7). PD-L1 distributed on tumor cells or tumor-infiltrating immune cells (TICs) can bind with the co-inhibitory molecule PD-1 on T cells and then promote T-cell exhaustion (8). Exhausted CD8+ T cells have significantly reduced cytotoxicity, which facilities the cancer cells escape from T cell-mediated immune surveillance (7, 9). These findings suggest that overexpressed PD-L1 may serve as a potential biomarker to predict the tumor progression, poor prognosis and therapeutic response. This hypothesis has been proved by meta-analyses on several cancers, including gynecological cancer types (10–12). For example, Gu et al., synthesized 7 studies of cervical cancer and found that PD-L1 overexpression was related with poor OS [hazard ratios (HR) = 2.52; 95% confidence interval (CI) =1.09 – 5.83, p = 0.031] in overall or Asian patients and progression-free survival (PFS) (HR = 4.78; 95% CI = 1.77–12.91, p = 0.002) only in Asian subgroup (10). This predictive significance of positive PD-L1 expression for shorter OS (HR = 1.66) and PFS (HR = 2.17) was also demonstrated in a meta-analysis for Asian patients with ovarian cancer (12). Lu et al. reported that PD-L1 expression was significantly associated with poor differentiation (odds ratios = 2.82) and advanced International Federation of Gynecology and Obstetrics (FIGO) stage (odds ratios = 1.71) of endometrial cancer patients (11). However, there was still no meta-analysis to integrate all gynecological cancer types. More importantly, the number of included publications was relatively fewer (all < 10) in these three published meta-analyses of each gynecological cancer type (10–12). Furthermore, the clinical association of PD-L1 was not analyzed for ovarian cancer previously (12); the association of PD-L1 to anti-PD-1/PD-L1 treatment was not investigated in any type; data of tumor cells and TICs were not both collected in endometrial and cervical cancer studies (10, 11) and thus their specific associations could not be performed. Hereby, the predictive performance of PD-L1 for patients with gynecological cancer remains inconclusive.

In the present study, we attempted to conduct an updated meta-analysis based on 55 published evidences to re-investigate the association of PD-L1 expression status in tumor cells and TICs with the prognosis, clinicopathological characteristics and response to anti-PD-1/PD-L1 treatment in gynecological cancer patients.

## Materials and Methods

This meta-analysis followed the guidelines of the Preferred Reporting Items for Systematic Review and Meta-analysis (PRISMA). Patient consent and ethical approval were waived because this study collected the data from published articles.

### Literature Search

The online databases of the PubMed, the Cochrane Library and Embase were systematically searched up to April, 2020. The following key words were applied for searches: (“gynecological” OR “cervical” OR “ovarian” OR “endometrial”) AND (“cancer” OR “carcinoma” OR “tumor”) AND (“PD-L1” OR “programmed death ligand-1” OR “B7-H1” OR “CD274”). The reference lists in the retrieved papers and relevant reviews were also checked to identify additional publications.

### Inclusion and Exclusion Criteria

Two reviewers independently evaluated potential articles. Studies which met the following inclusion criteria were considered eligible: 1) patients were diagnosed as any one type of gynecological cancers by pathological analyses (regardless of epithelial cancers, sarcomas or neuroendocrine tumors); 2) tumor samples for detection of PD-L1 expression were collected during primary tumor removal surgery or diagnostic biopsy before any treatment (such as neoadjuvant chemotherapy, PD-1/PD-L1 inhibitor); 3) the protein expression of PD-L1 on tumor cells or TICs of cancer tissues was determined using immunohistochemistry (IHC); 4) prognosis [OS, PFS, recurrence-free survival (RFS), cancer-specific survival (CSS) or disease-free survival (DFS)], clinicopathological parameters [FIGO stage, lymph node metastasis (LNM), tumor size, depth of infiltration, lymphovascular space invasion (LVSI), FIGO grade] and therapeutic response outcomes [objective response rate (ORR)] were compared between groups with high (positive) and low (negative) expression of PD-L1; 5) HR or relative risks (RR) as well as 95% CI values could be directly extracted, indirectly calculated using raw data or estimated from Kaplan–Meier curve; and 6) the studies were published in English and full-text. Studies were excluded if they were: 1) duplicate articles; 2) case reports, reviews, meeting abstracts, comments or letters; 3) studies evaluating the expression of PD-L1 at mRNA levels or at protein levels using other methods; 4) studies measuring the expression of PD-L1 after treatment; 5) studies having no usable data to estimate HRs and 95%CIs; 6) studies focusing on other cancers; and 7) studies written in other languages. Any disagreements were solved by discussion.

### Data Extraction and Quality Assessment

Two researchers independently extracted the following data from each study: name of the first author, year of publication, country, population number, cancer type, clinicopathological features, prognostic endpoint, treatment, IHC detection area/antibody type/antibody source/IHC counting method/cut-off point for PD-L1, HRs with 95% CIs and their statistical analysis approach. Multivariable analysis results were preferentially extracted to obtain HRs and 95%CIs; otherwise, univariate analysis results were collected. The survival data in the Kaplan-Meier curves were read using a digitizing software-Engauge Digitizer 4.1. Any disputes were resolved through discussion.

The quality of included studies was assessed using the Newcastle-Ottawa Scale (NOS) (13) that consists of three key domains: selection, comparability and outcomes or exposure. Total NOS score ranged from 0 to 9. Studies with the final score > 6 were considered to have a high methodological quality.

### Statistical Analysis

All data analyses were achieved with STATA 13.0 software (STATA Corporation, College Station, TX, USA). HRs with 95% CIs from each study were pooled to determine the association of PD-L1 expression with the prognostic indicators; while RRs with 95% CIs were utilized to measure the correlation of PD-L1 expression with clinicopathological factors and ORR. HR or RR > 1 indicated a poorer prognosis or higher degree of malignancy in patients with high PD-L1 expression. Association difference was analyzed using z test (p < 0.05). Heterogeneity across studies was quantified by using the Q-test and I^2^ statistic. P < 0.10 and I^2^ > 50% were set as the threshold for defining the studies with significant heterogeneity. A random-effect model was chosen to compute the pooled HR (or RR) for variables from studies with heterogeneity. A fixed-effect model was adopted for studies without evidence of heterogeneity. Egger’s linear regression test (14) was used to detect the publication bias. If bias was seen (p < 0.05), “trim and fill” algorithm (15) was chosen for adjustment of HRs (RRs). Subgroup analysis was also carried out according to study country, sample size, cancer type, IHC detection area, antibody type, antibody source, IHC counting method, cut-off value, HR source and statistical approach to investigate possible causes of heterogeneity. Sensitivity analysis was performed *via* omitting any one study at a time. *P*-values and 95% CIs were two-sided.

## Results

### Study Selection

**Figure 1:**
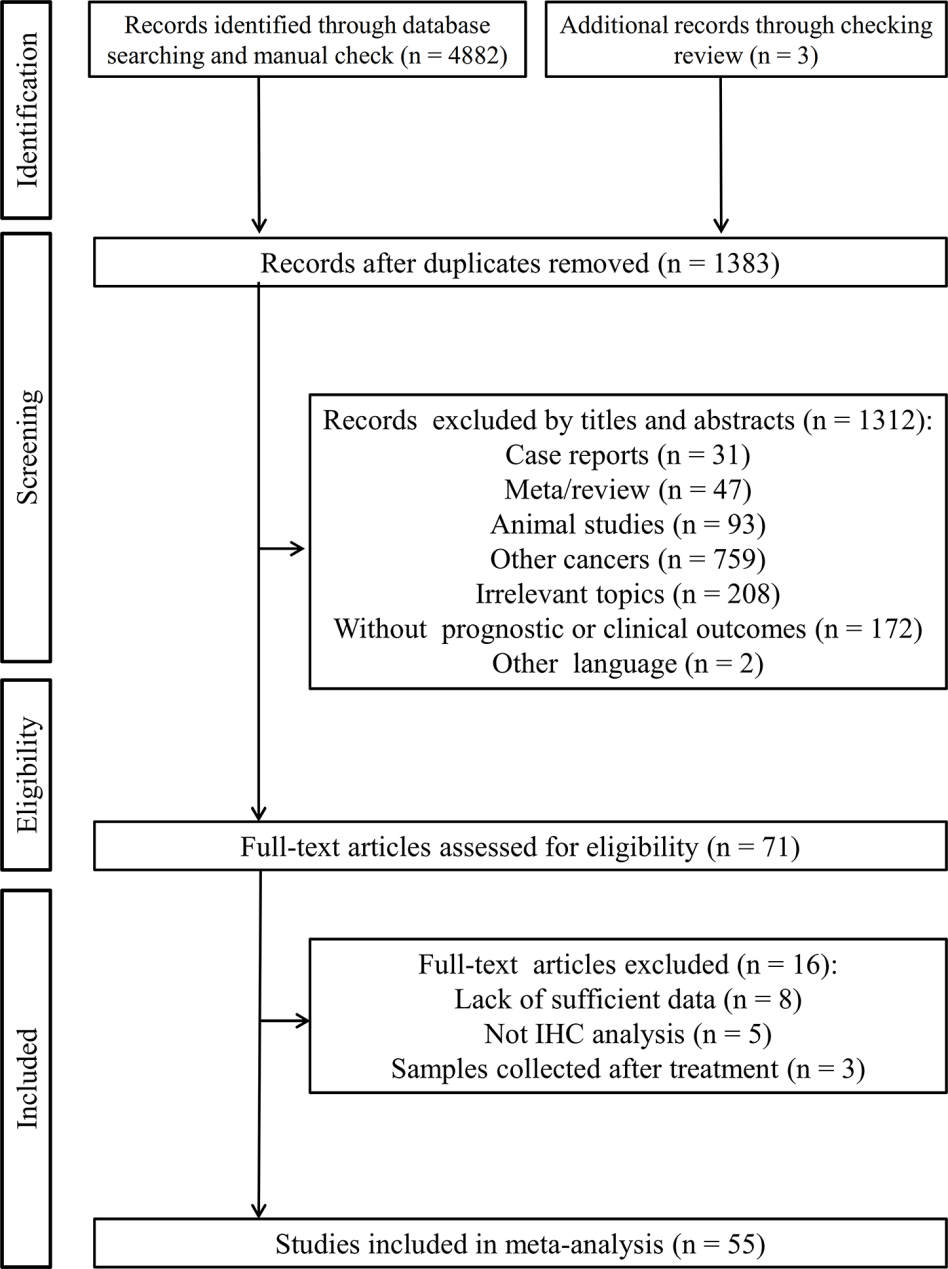
Flowchart of the study inclusion process.

**Figure 1** outlines the flowchart of the literature collection process. A total of 4,882 records were initially identified through searching the electronic database. After removal of 3,502 duplicate records, the titles and abstracts of 1,380 studies were read. Consequently, 1,312 articles were excluded because of they were: case reports (n = 31), meta/review (n = 47), animal studies (n = 93), studies investigating other cancers (n = 759), irrelevant topics (n = 208), without survival or other clinical outcomes (n = 172) and published in other languages (n = 2). After reviewing 68 full-text articles in detail, 16 studies were further removed since sufficient data were not provided for analysis (n = 8), IHC method was not used for detection of PD-L1 protein expression (n = 5) or the samples were collected after treatment (n = 3). Additional 3 studies were supplemented through checking the references of reviews. Finally, 55 studies were eligible for the meta-analysis (16–70).

### Characteristics of the Included Studies

**Table 1** shows the characteristics of all the included studies. The publication years ranged from 2007 to 2020 and 61.8% (34/55) of them were published within 2019 and 2020. Fourteen studies were performed in China, nine were in the USA, eight were in Japan, four in Korea, each three in Thailand, Turkey, each two in Canada, France, Germany and each one in Norway, Belgium, Brazil, Denmark, Egypt, Greece, Sweden and UK. Twenty-three studies explored the association of PD-L1 with clinical outcomes in ovarian cancer patients, 15 focused on cervical cancer and 14 investigated endometrial cancer. Ovarian and endometrial cancer patients were both enrolled in two studies, while cervical and endometrial cancer patients were both collected in one study. The prognostic endpoint was OS in 38 studies, PFS in 20 studies, RFS in 2 studies, CSS in 6 studies and DFS in 5 studies. FIGO stage (II-IV vs I or III-IV vs I-II) was compared between the groups with high and low expression of PD-L1 in 27 studies; tumor size (≥40 mm vs < 40 mm) was described in 5 studies; LNM (yes vs no) was reported in 16 studies; infiltration depth (≥ 1/2 vs <1/2) was analyzed in 7 studies; LVSI (yes vs no) was observed in 14 studies; FIGO grade was explored in 13 studies. One thing should be noted that tumor cells and TICs were both analyzed and the different IHC counting methods (cut-off points) were applied in some studies, which led to more datasets used for analysis of the prognostic and clinical significance of PD-L1 compared with the actual number of papers (**Table S1**). The patients in most of these studies underwent surgery, radiotherapy and/or chemotherapy with routine drugs, while six studies specifically explored the efficacy of anti-PD-1/PD-L1 antibodies (pembrolizumab, atezolizumab, nivolumab) for the treatment of gynecological cancers (65–70). The association of PD-L1 expression status with ORR, OS and PFS to these anti-PD-1/PD-L1 immune checkpoint inhibitors was also investigated in these six studies (65–70). The NOS scores of all included studies were > 6, suggesting the methodological quality was high for all of them (**Table S2**).

**Table 1:** Characteristics of included studies.

Study	Year	Country	No.	Cancer type	Clinical endpoint	Clinicopathological factors	HR for survival analysis		PD-L1 expression		
							Calculation method	Source	Detected area by IHC	IHC counting method	Cut-off value
Wang S (16)	2018	China	90	CC	OS, PFS	LVSI, FIGO stage, infiltration depth, LNM, tumor size	UV	Reported	Tumor cells	IRS	H-score of 100
Enwere EK (17)	2017	Canada	120	CC	OS, PFS	FIGO stage, LNM	UV	Reported	Tumor cells	SP, SI	Median percentage, Median tAQUA score
Feng M (18)	2018	China	219	CC	OS	LVSI, infiltration depth; LNM, grade, tumor size	UV	Estimated	Tumor cells, TICs	SP	>5%
Kim M (19)	2017	Korea	27	CC	OS, PFS	LVSI, LNM	UV	Estimated	Tumor cells	SP	>1%
Iijima M (20)	2020	Japan	33	CC	OS, PFS	FIGO stage, LNM	UV	Estimated	Tumor cells	SP	>1%
Tsuchiya T (21)	2020	Japan	104	CC	OS	FIGO stage, LNM	UV	Reported	Tumor cells, TICs	SI	Score (tumor cells, 0; TICs, 3)
Kawachi A (22)	2018	Japan	148	CC	DFS	LVSI, LNM, tumor size	UV	Estimated	Tumor cells	SP	>5%
Loharamtaweethong K (23)	2019	Thailand	171	CC	RFS, CSS		UV (CSS), MV (RFS)	Reported	Tumor cells	SP	>5%
Miyasaka Y (24)	2020	Japan	71	CC	OS, PFS		MV	Reported	Tumor cells	SP	>1%
Chen H (25)	2020	China	222	CC	OS, DFS		MV	Reported	Tumor cells, TICs	SP	Tumor cells, >1%; TICs, >5%
Lippens (26)	2020	Belgium	38	CC	CSS		UV	Estimated	TICs	SP	>5%
Karim R (27)	2009	USA	115	CC	OS	LVSI, LNM, tumor size	UV	Estimated	Tumor cells	SP	>0%
Loharamtaweethong K (28)	2019	Thailand	153	CC	RFS, CSS	FIGO stage, LNM, tumor size	UV	Estimated	Tumor cells	SP	>10%
Grochot RM (29)	2019	Brazil	59	CC	OS, PFS		UV	Estimated	Tumor cells	SP	>0%
Xu M (30)	2016	China	112	OC		FIGO stage, grade			Tumor cells	IRS	Score > 4
Nhokaew W (31)	2019	Thailand	92	OC	DFS		UV	Estimated	Tumor cells	SI	Score > 2
Schmoeckel E (32)	2019	Germany	288	OC	OS		MV	Reported	Tumor cells	SP	>1%
Hamanishi J (33)	2007	Japan	50	OC	OS, PFS	FIGO stage, LNM	MV	Reported	Tumor cells	SI	Score > 1
Mesnage SJL (34)	2017	France	50	OC	PFS		UV	Reported	TICs	SP	>5%
Zhu J (35)	2017	China	122	OC	OS, PFS	FIGO stage	MV	Reported	Tumor cells	SP	>10%
Zhu J (36)	2017	China	19	OC	OS	FIGO stage	UV	Estimated	Tumor cells	SP	>10%
Zong L (37)	2020	China	146	OC	OS, PFS		UV	Estimated	CP	SP	>1%
Wang Q (38)	2017	China	107	OC	OS	FIGO stage	MV (tumor cells), UV (TICs)	Reported	Tumor cells, TICs	SP	>5%
Zhu X (39)	2018	China	112	OC	OS	FIGO stage, LNM, grade	UV	Estimated	Tumor cells	SP (or SI)	>10% (or score > 1)
Buderath P (40)	2019	Germany	179	OC	OS		UV	Estimated	TICs	SP	>0%
Kim KH (41)	2019	Korea	248	OC	OS	FIGO stage, grade	MV	Reported	Tumor cells, TIC	SP + SI	>5% + score > 2
Zhu X (42)	2019	China	112	OC	OS, DFS	FIGO stage, LNM, grade	UV	Reported	Tumor cells	SP (or SI)	>10% (or score > 1)
Zhang L (43)	2019	China	124	OC	OS, PFS	FIGO stage, LNM, grade	MV	Reported	Tumor cells	IRS	Score > 3
Alldredge J (44)	2019	USA	46	OC/EC	OS	FIGO stage	UV	Reported	Tumor cells, TIC	Tumor cells, SP; CPS, IRS	Tumor cells, >0%; CPS, score > 1
De La Motte Rouge T (45)	2019	France	51	OC	OS, DFS		UV	Reported	Tumor cells	Other	> 1000
Martin de la Fuente L (46)	2020	Sweden	130	OC	OS		MV	Reported	TICs	SP	> 1%
Chatterjee J (47)	2017	UK	48	OC	PFS		UV	Reported	TICs	SI	Median score
Henriksen JR (48)	2020	Denmark	283	OC	OS	FIGO stage	MV	Reported	Tumor cells	SP	> 1%
Sungu N (49)	2019	Turkey	127	EC	OS	LVSI, FIGO stage, grade	UV	Estimated	Tumor cells, TICs	SI + SP	Score > 2 (≥ 1%)
Vagios S (50)	2019	Greece	101	EC	OS, PFS	LVSI, FIGO stage, infiltration depth, LNM	MV	Reported	Tumor cells	SP	> 1%
Kucukgoz Gulec U (51)	2019	Turkey	53	EC	OS		MV	Reported	Tumor cells	SP	> 5%
Zhang S (52)	2020	Japan	221	EC	OS	LVSI, FIGO stage, infiltration depth, FIGO grade	MV	Reported	Tumor cells, TICs	IRS, SI	TC, score > 0; TICs, score > 4
Kim J (53)	2018	Korea	183	EC	OS, PFS	LVSI, FIGO stage, infiltration depth, grade	UV (tumor cells), MV (TICs)	Reported	Tumor cells, TICs	SI	> 1.977
Jones TE (54)	2021	USA	43	EC	OS	FIGO stage	UV	Reported	CP	SP	> 5%
Kucukgoz Gulec U (55)	2020	Turkey	59	EC	OS		MV	Reported	Tumor cells	SP	>5%
Tawadros AIF (56)	2018	Egypt	95	EC		LVSI, FIGO stage, infiltration depth, LNM, grade			Tumor cells	IRS	Score >3
Li ZB (57)	2017	USA	700	EC	CSS	LVSI	UV	Estimated	Tumor cells, TICs	SP	>1%
Mo ZF (58)	2016	China	75	EC		LVSI, FIGO stage			Tumor cells, TICs	IRS	>5%
Yamashita H (59)	2018	Japan	149	EC	OS, PFS		UV	Estimated	Tumor cells	SP	>5%
Engerud H (60)	2020	Norway	700	EC	CSS	FIGO stage, infiltration depth, grade	UV	Estimated	Tumor cells	IRS	Score >0
Crumley S (61)	2019	USA	132	EC		LVSI, FIGO stage, infiltration depth, LNM			Tumor cells	SI + SP	Score >2 + ≥ 0%; Score >3 + > 2%
Li MJ (62)	2017	China	113	OC	OS	FIGO stage	UV (DFS), MV (OS)	Reported	Tumor cells	IRS	Score >2
Webb JR (63)	2016	Canada	479	OC	CSS	FIGO stage, grade	MV (HGSC), UV (other)	Reported, estimated	CP	SI	Score >1
Xue CY (64)	2020	China	77	OC	OS, PFS	FIGO stage, grade	MV (OS), UV KM (PFS)	Reported	Tumor cells	IRS	H-score of 100
Chung HC (65)	2019	Korea	98	CC	OS, PFS, ORR		UV	Estimated	CP	SI	Score >1
Liu JF (66)	2019	USA	12/15	OC/EC	OS, PFS, ORR		UV	Estimated	TICs	SI	Score >1
Matulonis UA (67)	2019	USA	338	OC	ORR				CP	SI	Score >1
Zamarin D (68)	2020	USA	52	OC	PFS, ORR		UV	Reported	Tumor cells, TICs	SP	Tumor cells, > 1%; TICs, > 1% or 10%
Santin AD (69)	2020	USA	22	EC	ORR				CP	SI	score >1
Tamura K (70)	2019	Japan	44	CC,EC	OS, PFS, ORR		UV	Estimated	Tumor cells	SP	>1%

OS, overall survival; PFS, progression free survival; RFS, recurrence-free survival; CSS, cancer-specific survival; DFS, disease-free survival; FIGO, International Federation of Gynecology and Obstetrics; LNM, lymph node metastasis; LVSI, lymphovascular space invasion; ORR, overall response rate; KM, Kaplan–Meier curve; UV, univariate analysis; MV, multivariate analysis; SP, staining percentage; SI, staining intensity score; IRS, immunoreactive SI (that is, IRS = SI × SP); IHC, immunohistochemistry; TICs, tumor-infiltrating immune cells; CPS, combined positive; estimated, the HR was obtained from Kaplan–Meier curve; HGSC, high-grade serous ovarian cancer.

### Association Between Programmed Cell Death-Ligand 1 Expression and Survival

#### Overall Analysis in All Gynecological Cancers

Fifty-one datasets (**Table S1**) reported the predictive values of PD-L1 for OS in all gynecological cancers. The random-effects model was chosen because of significant heterogeneity (I^2 ^= 71.7%, p = 0.000). The results of the meta-analysis indicated no significant association of PD-L1 expression with OS (HR = 1.13; 95% CI: 0.91 – 1.39, p = 0.263). Data on PFS were extracted from 26 datasets (**Table S1**). The pooled results showed that PD-L1 expression was not significantly associated with PFS (HR = 1.04; 95% CI: 0.85 – 1.29, p = 0.682) under a random-effect model (I^2^ = 63.7%, p = 0.000). Meta-analysis using the corresponding datasets also demonstrated that positive expression of PD-L1 was not related to RFS (n = 2; HR = 1.08; 95% CI: 0.64 – 1.83, p = 0.778; I^2^ = 0%, p = 0.746), DFS (n = 6: HR = 1.26; 95% CI: 0.60 – 2.64, p = 0.545; I^2^ = 81.5%, p = 0.000) and CSS (n = 10: HR = 0.81; 95% CI: 0.65 – 1.01, p = 0.056; I^2^ = 28.8%, p = 0.180).

#### Subgroup Analysis in All Gynecological Cancers

**Figure 2:**
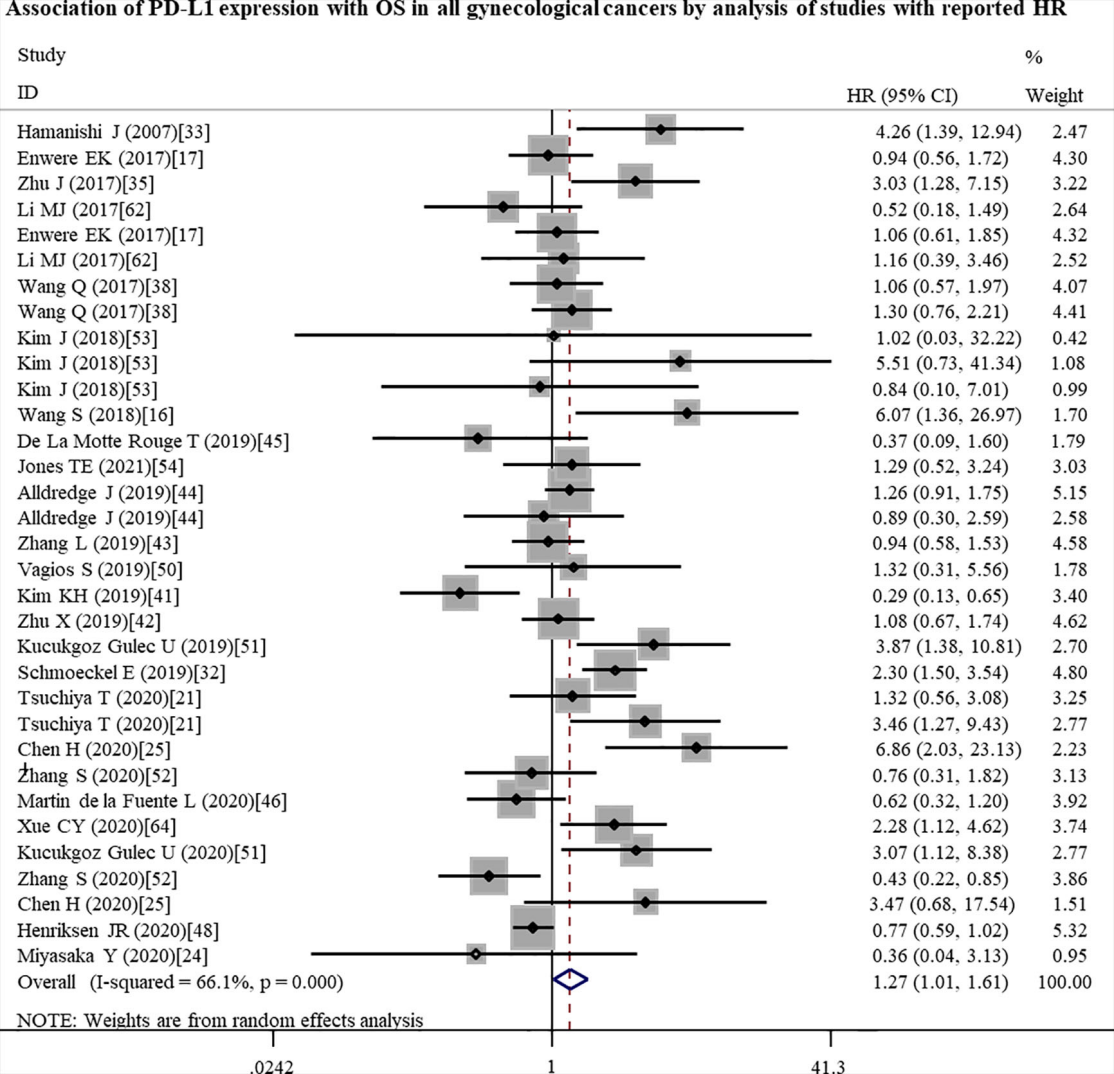
Forest plots showing the significant association between high PD-L1 expression and a poor overall survival (OS) in all gynecological cancers patients by analysis of the studies with reported HR. HR, hazard ratio; CI, confidence interval.

**Figure 3:**
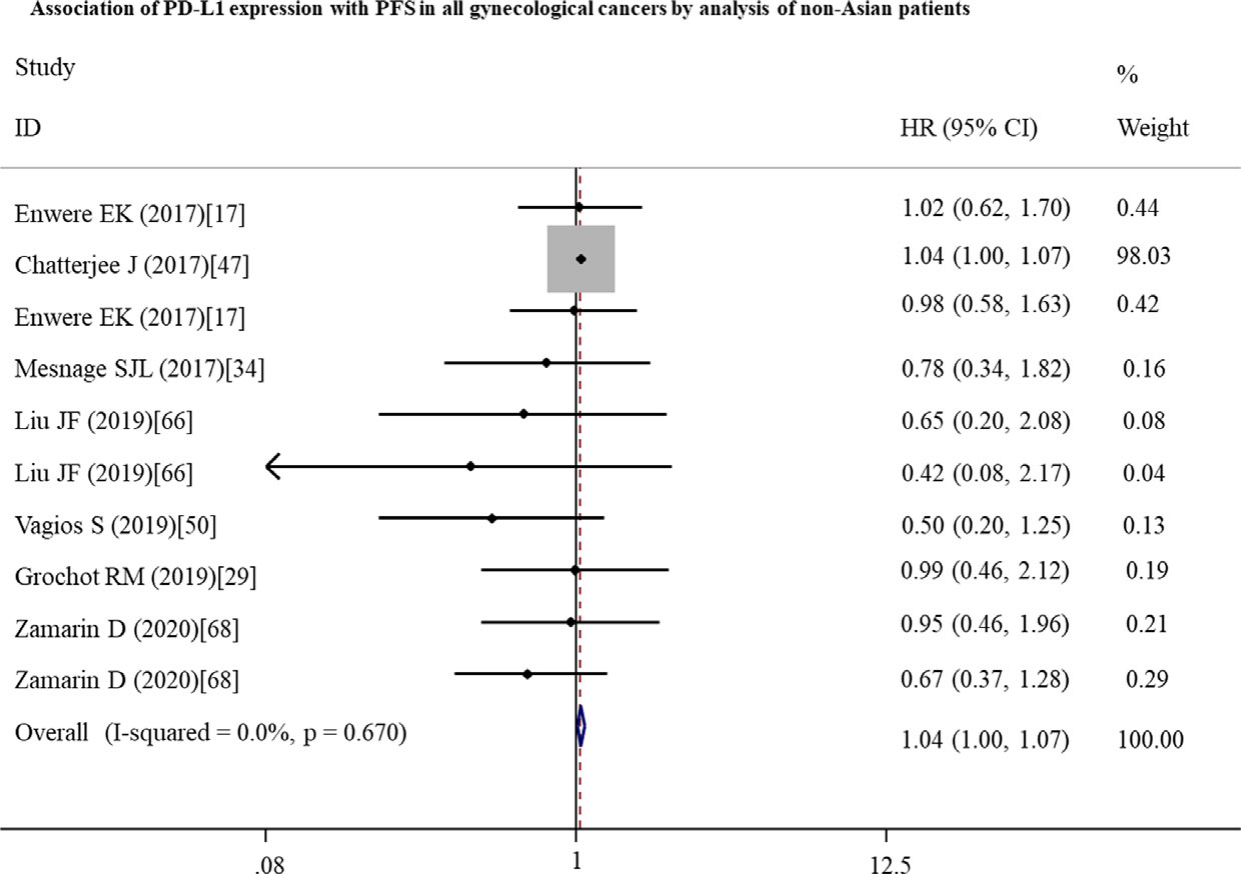
Forest plots showing the significant association between PD-L1 expression and a shorter progression-free survival (PFS) in all gynecological cancers patients from non-Asian countries. HR, hazard ratio; CI, confidence interval.

To further investigate the possible prognostic potential of PD-L1 in gynecological cancers, the subgroup analysis was performed. The results showed that, in studies with reported HR, high PD-L1 expression was correlated with shorter OS (n = 33: HR = 1.27; 95% CI: 1.01 – 1.61, p = 0.041) (**Figure 2**; **Table 2**). Furthermore, PD-L1-positive status with a cut-off value of 5% predicted a poor OS (n = 8: HR = 2.10; 95% CI: 1.17 – 3.75, p = 0.013), but not 1% or others (**Table 2**). Although a significant association between PD-L1 and PFS was also observed in analyses of non-Asian population (n = 10: HR = 1.04; 95% CI: 1.00 – 1.07, p = 0.040) (**Figure 3**; **Table 3**), the corresponding HR was relatively lower and approximated to 1, indicating the clinical relevance of PD-L1 expression with PFS may be insignificant. The conclusions of PFS from estimated HR may be undetermined, although it was significant (p = 0.001). Owing to the small number of included studies, subgroup analysis was not performed for RFS, DFS and CSS.

**Table 2:** Subgroup analysis on the outcome of OS.

Comparison		Studies	HR(95%CI)	*P_z_-*value	I^2^	*P_H_*-value
Region	Asian	32	1.22(0.88,1.68)	0.237	76.1	0.000
	Non-Asian	19	1.05(0.81,1.37)	0.699	61.5	0.000
Sample size	<100	20	1.09(0.72,1.64)	0.694	73.2	0.000
	>100	31	1.15(0.90,1.48)	0.265	71.5	0.000
IHC counting method	SI	12	0.97(0.52,1.79)	0.922	72.7	0.000
	SP	32	1.21(0.93,1.56)	0.158	72.5	0.000
	IRS	6	1.14(0.69,1.89)	0.598	72.5	0.003
	Other	1	0.37(0.09,1.56)	0.176	–	–
Cut-off values	1%	13	0.96(0.66,1.46)	0.939	60.7	0.002
	5%	8	2.10(1.17,3.75)	**0.013**	75.9	0.000
	Others	30	0.99(0.77,1.28)	0.949	67.1	0.000
Cancer type	Ovarian	22	1.02(0.80,1.30)	0.884	69.9	0.000
	Cervical	16	1.31(0.76,2.27)	0.338	81.3	0.000
	Endometrial	13	1.23(0.77,1.98)	0.381	50.0	0.020
Antibody type	Monoclonal	48	1.09(0.87,1.36)	0.447	71.8	0.000
	Unclear	3	1.94(0.77,4.88)	0.161	75.2	0.018
Antibody source	Mouse	8	1.27(0.64,2.54)	0.495	81.9	0.000
	Rabbit	40	1.07(0.85,1.35)	0.566	69.0	0.000
	Unclear	3	1.94(0.77,4.88)	0.161	75.2	0.018
IHC detection area	Tumor cells	31	1.32(0.99,1.74)	0.052	69.6	0.000
	TICs	16	0.94(0.66,1.34)	0.751	63.5	0.000
	Tumor cells + TICs	4	0.75(0.34,1.63)	0.463	85.0	0.000
HR method	MV	21	1.34(0.94,1.91)	0.103	75.3	0.000
	UV	30	1.01(0.77,1.32)	0.958	69.5	0.000
HR source	Reported	33	1.27(1.01,1.61)	**0.041**	66.1	0.000
	Estimated	18	0.86(0.55,1.35)	0.513	78.2	0.000

OS, overall survival; UV, univariate analysis; MV, multivariate analysis; SP, staining percentage; SI, staining intensity score; IRS, immunoreactive SI (that is, IRS = SI × SP); HR, hazard ratio; CI, confidence interval; IHC, immunohistochemistry; TICs, tumor-infiltrating immune cells. P_Z_, p-value for association; P_H_, p-value for heterogeneity obtained by Q-test; I^2^, the degree of heterogeneity by I^2^ statistic. Bold indicated the significance after analysis of two or more than two studies (p < 0.05).

**Table 3:** Subgroup analysis on the outcome of PFS.

Comparison		Studies	HR(95%CI)	*P_z_-*value	I^2^	*P_H_*-value
Region	Asian	16	1.30(0.86,1.97)	0.209	75.8	0.000
	Non-Asian	10	1.04(1.00,1.07)	**0.040**	0.0	0.670
Sample size	<100	16	0.98(0.72,1.34)	0.921	70.4	0.000
	>100	10	1.14(0.83,1.56)	0.423	50.4	0.033
IHC counting method	SI	8	1.22(0.73,2.05)	0.451	77.3	0.000
	SP	15	0.89(0.74,1.08)	0.226	0.0	0.478
	IRS	3	2.22(0.75,6.53)	0.149	87.7	0.000
Cut-off values	1%	8	0.75(0.55,1.02)	0.065	0.0	0.669
	5%	2	0.76(0.43,1.36)	0.361	0.0	0.947
	Others	16	1.25(0.94,1.65)	0.120	74.4	0.000
Cancer type	Ovarian	10	1.14(0.87,1.49)	0.360	62.0	0.005
	Cervical	9	0.87(0.54,1.39)	0.561	68.6	0.001
	Endometrial	7	1.27(0.70,2.30)	0.431	56.1	0.034
Antibody type	Monoclonal	22	0.95(0.73,1.22)	0.665	52.1	0.002
	Unclear	4	1.65(0.90,3.01)	0.106	86.3	0.000
Antibody source	Mouse	3	0.79(0.26,2.41)	0.684	85.7	0.001
	Rabbit	19	0.99(0.79,1.24)	0.894	28.5	0.120
	Unclear	4	1.65(0.90,3.01)	0.106	86.3	0.000
IHC detection area	Tumor cells	17	1.16(0.86,1.56)	0.337	59.8	0.001
	TICs	7	1.05(0.68,1.61)	0.830	54.4	0.041
	Tumor cells + TICs	2	0.60(0.29,1.24)	0.167	75.7	0.043
HR method	MV	7	1.46(0.82,2.62)	0.201	68.4	0.004
	UV	19	0.95(0.76,1.20)	0.661	62.3	0.000
HR source	Reported	16	1.29(1.00,1.67)	0.052	67.1	0.000
	Estimated	10	0.65(0.50,0.84)	**0.001**	3.3	0.409

OS, overall survival; UV, univariate analysis; MV, multivariate analysis; SP, staining percentage; SI, staining intensity score; IRS, immunoreactive SI (that is, IRS = SI × SP); HR, hazard ratio; CI, confidence interval; IHC, immunohistochemistry; TIC, tumor-infiltrating immune cells. P_Z_, p-value for association; P_H_, p-value for heterogeneity obtained by Q-test; I^2^, the degree of heterogeneity by I^2^ statistic. Bold indicated the significance after analysis of two or more than two studies (p < 0.05).

#### Sub-Subgroup Analysis in Each Cancer Type

**Figure 4:**
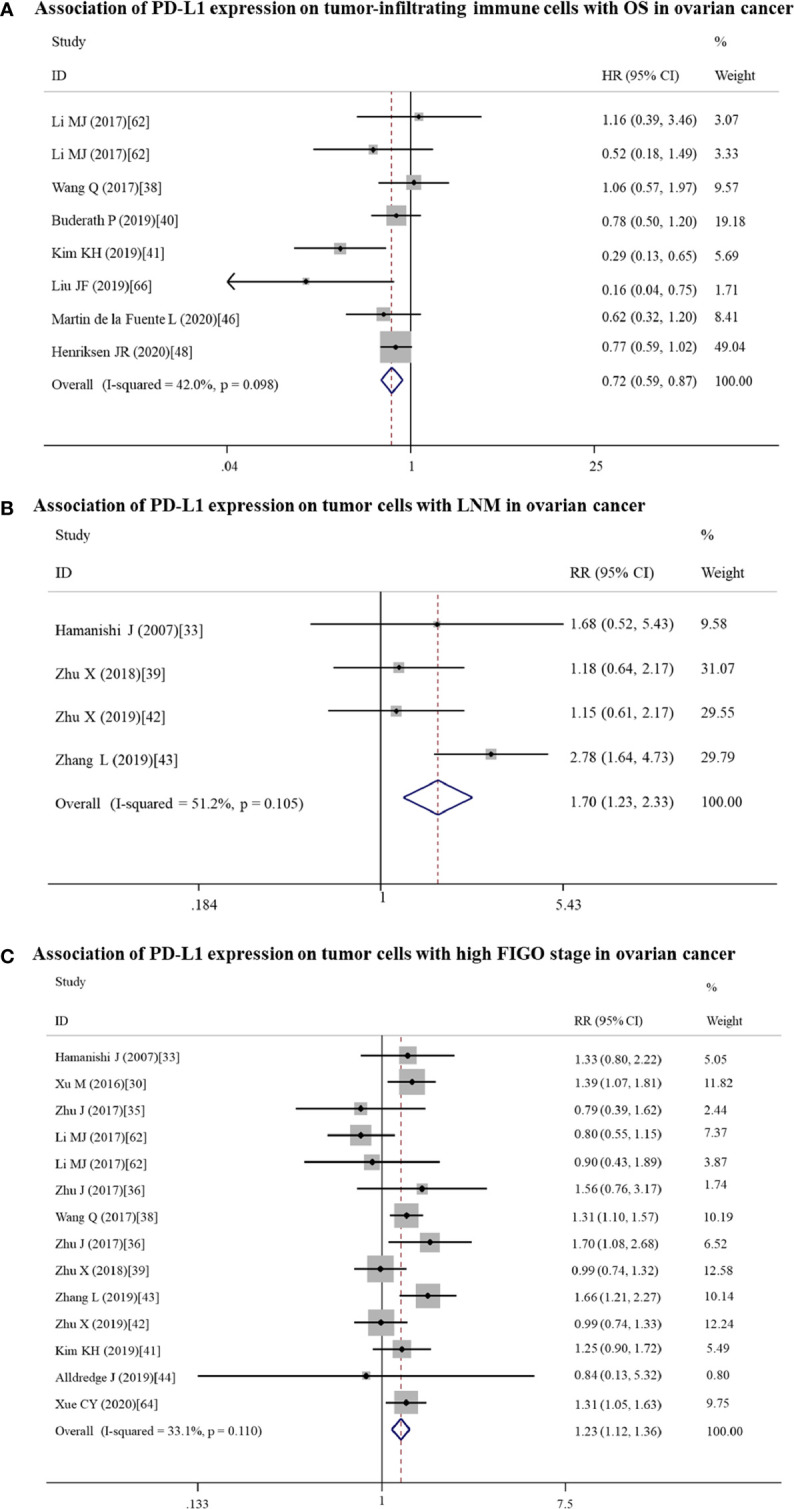
Forest plots showing the association of PD-L1 expression for ovarian cancer patients. **(A)** PD-L1 expression on tumor-infiltrating immune cells and overall survival (OS). **(B)** PD-L1 expression on tumor cells and LNM. **(C)** PD-L1 expression on tumor cells and FIGO stage. FIGO, International Federation of Gynecology and Obstetrics; LNM, lymph node metastasis; HR, hazard ratio; RR, relative risk; CI, confidence interval.

In addition, non-significant relationships were seen between PD-L1 and OS/PFS in any type of gynecological cancers (**Tables 2** and **3**). To further explore whether PD-L1 expression may be a significant prognostic factor for specific gynecological cancer type, the sub-subgroup analysis was also conducted. The results revealed that PD-L1 overexpression on TICs predicted a favorable OS for ovarian cancer (n = 8: HR = 0.72; 95% CI: 0.59 – 0.87, p = 0.001; **Figure 4A**); while predicted a shorter OS for cervical cancer patients (n = 3: HR = 3.44; 95% CI: 1.78 – 6.66, p = 0.000) (**Table S3**). Also, the positive association between PD-L1 expression and OS in cervical cancer patients was proved in studies with reported HR (n = 8: HR = 1.89; 95% CI: 1.06 – 3.36, p = 0.031) and sample size > 100 (n = 9: HR = 1.92; 95% CI: 1.07 – 3.45, p = 0.030), further increasing the credibility to use PD-L1 as the prognostic biomarker for cervical cancer (**Table S3**). Likewise, PD-L1 overexpression was correlated with a lower OS rate in non-Asian individuals with endometrial cancer (n = 7: HR = 1.60; 95% CI: 1.07 – 2.40, p = 0.022) (**Table S3**). The cut-off value of 5% may be optimal (n = 3: HR = 2.37; 95% CI: 1.35 – 4.18, p = 0.003) compared with 1% and others (**Table S3**). The association between PD-L1 expression and PFS may be clinically significant only in the Asian endometrial cancer patients (n = 5: HR = 1.59; 95% CI: 1.01 – 2.51, p = 0.045) (**Table S4**), but not in cervical cancer because the pooled HR was obtained from estimated HR in most of individual studies (**Table S1**) or in ovarian cancer because the pooled HR approximated to 1 (**Table S4**).

### Association of Programmed Cell Death-Ligand 1 Expressions With Clinicopathological Characteristics

#### Overall Analysis in All Gynecological Cancers

As shown in **Table 4**, the overall pooled results showed that PD-L1 overexpression correlated with LNM (n = 21: RR = 1.23; 95% CI = 1.09 – 1.51, p = 0.003), advanced FIGO stage (III–IV vs I-II) (n = 34: RR = 1.18; 95% CI = 1.05 – 1.32, p = 0.007) and LVSI (n = 20: RR = 1.26; 95% CI = 1.05 – 1.57, p = 0.034).

**Table 4:** Correlations between PD-L1 expression and clinical characteristics.

Comparison			Studies	RR(95%CI)	*P*-value	I^2^	*P*-value
LNM (yes vs no)	Overall		21	1.23(1.09,1.51)	**0.003**	42.2	0.022
	Cancer type	Ovarian	4	1.70(1.23,2.34)	**0.001**	51.2	0.105
		Cervical	11	1.03(0.83,1.27)	0.792	29.3	0.167
		Endometrial	6	1.85(1.17,2.91)	**0.008**	46.3	0.097
	IHC detection area (overall)	Tumor cells	19	1.33(1.12,1.59)	**0.001**	42.5	0.027
		TICs	2	0.98(0.64,1.49)	0.907	48.2	0.165
	IHC detection area (ovarian)	Tumor cells	4	1.70(1.23,2.33)	**0.001**	51.2	0.105
	IHC detection area (cervical)	Tumor cells	9	1.05(0.82,1.33)	0.725	33.7	0.148
		TICs	2	0.98(0.64,1.49)	0.907	48.2	0.165
	IHC detection area (endometrial)	Tumor cells	6	1.85(1.17,2.91)	**0.008**	46.3	0.097
Tumor size (≥4 cm vs < 4 cm)	Overall		6	1.05(0.86,1.29)	0.637	23.7	0.256
	Cancer type	Cervical	6	1.05(0.86,1.29)	0.637	23.7	0.256
	IHC detection area (overall)	Tumor cells	5	1.11(0.90,1.37)	0.339	10.6	0.346
		TICs	1	0.61(0.24,1.51)	0.283	–	–
FIGO stage (III-IV vs I-II)	Overall		34	1.18(1.05,1.32)	**0.007**	55.0	0.000
	Cancer type	Ovarian	21	1.14(1.01,1.29)	**0.039**	57.7	0.001
		Cervical	2	1.85(0.97,3.54)	0.061	0.0	0.764
		Endometrial	11	1.30(0.95,1.77)	0.106	53.3	0.018
	IHC detection area (overall)	Tumor cells	23	1.21(1.07,1.37)	**0.003**	42.5	0.017
		TICs	4	1.22(0.85,1.76)	0.279	82.5	0.001
		Tumor cells + TICs	7	0.89(0.65,1.22)	0.470	0.0	0.656
	IHC detection area (ovarian)	Tumor cells	14	1.23(1.12,1.36)	**0.000**	33.1	0.110
		TICs	2	0.94(0.86,1.04)	0.254	66.5	0.084
		Tumor cells + TICs	5	0.82(0.56,1.19)	0.295	0.0	0.412
	IHC detection area (cervical)	Tumor cells	2	1.85(0.97,3.54)	0.061	0.0	0.764
	IHC detection area (endometrial)	Tumor cells	7	1.10(0.88,1.37)	0.412	60.5	0.019
		TICs	2	1.72(1.16,2.54)	**0.007**	0.0	0.605
		Tumor cells + TICs	2	0.98(0.54,1.77)	0.928	0.0	0.736
FIGO stage (II-IV vs I)	Overall		8	1.34(0.83, 2.16)	0.233	81.0	0.000
	Cancer type	Endometrial	4	2.90(1.70,4.94)	**0.000**	0.0	0.688
		Cervical	4	0.87(0.57,1.34)	0.520	79.1	0.002
	IHC detection area (overall)	Tumor cells	5	1.33(0.71,2.47)	0.371	85.5	0.000
		TICs	3	1.77(0.45,6.96)	0.417	78.6	0.009
	IHC detection area (cervical)	Tumor cells	3	0.89(0.71,1.12)	0.336	85.6	0.001
		TICs	1	0.73(0.51,1.05)	0.093	–	–
	IHC detection area (endometrial)	Tumor cells	2	2.96(1.58,5.55)	**0.001**	0.0	0.454
		TICs	2	3.47(1.23,9.83)	**0.019**	0.0	0.340
Infiltration depth (≥ 1/2 vs <1/2)	Overall		9	1.27(0.99,1.63)	0.058	78.1	0.000
	Cancer type	Cervical	1	1.12(0.96,1.30)	0.150	–	–
		Endometrial	8	1.34(0.96,1.87)	0.082	80.8	0.000
	IHC detection area (overall)	Tumor cells	7	1.15(0.88,1.49)	0.316	76.3	0.000
		TICs	2	1.77(1.33,2.35)	**0.000**	0.0	0.852
	IHC detection area (endometrial)	Tumor cells	6	1.03(0.89,1.19)	0.692	79.4	0.000
		TICs	2	1.77(1.33,2.35)	**0.000**	0.0	0.852
LVSI (yes vs no)	Overall		20	1.26(1.02,1.57)	**0.034**	69.5	0.000
	Cancer type	Cervical	6	0.91(0.77,1.09)	0.296	0.0	0.450
		Endometrial	14	1.51(1.15,2.00)	**0.004**	68.2	0.000
	IHC detection area (overall)	Tumor cells	13	1.25(0.95,1.64)	0.118	70.4	0.000
		TICs	6	1.41(0.95,2.10)	0.092	64.6	0.015
		Tumor cells + TICs	1	0.92(0.58,1.44)	0.700	–	–
	IHC detection area (cervical)	Tumor cells	5	0.92(0.76,1.11)	0.373	8.8	0.356
		TICs	1	0.80(0.50,1.28)	0.354	–	–
	IHC detection area (endometrial)	Tumor cells	8	1.61(1.03,2.51)	**0.035**	75.4	0.000
		TICs	5	1.71(1.34,2.18)	**0.000**	19.2	0.293
		Tumor cells + TICs	1	0.92(0.58,1.44)	0.700	–	–
Grade (G3 vs G1+ G2)	Overall		18	1.20(0.96,1.51)	0.111	74.0	0.000
	Cancer type	Ovarian	10	1.22(0.90,1.64)	0.205	66.8	0.001
		Cervical	2	0.88(0.76,1.01)	0.075	0.0	0.557
		Endometrial	7	1.48(0.79,2.77)	0.221	77.5	0.000
	IHC detection area (overall)	Tumor cells	11	1.01(0.76,1.35)	0.924	68.1	0.001
		TICs	5	1.86(0.99,3.47)	0.053	84.3	0.000
		Tumor cells + TICs	4	1.15(0.95,1.39)	0.145	0.0	0.806
	IHC detection area (ovarian)	Tumor cells	6	0.96(0.77,1.20)	0.722	24.2	0.252
		TICs	1	2.45(1.69,3.57)	0.000	–	–
		Tumor cells + TICs	4	1.15(0.95,1.39)	0.145	0.0	0.806
	IHC detection area (cervical)	Tumor cells	1	0.85(0.72,1.01)	0.070	–	–
		TICs	1	0.94(0.72,1.22)	0.629	–	–
	IHC detection area (endometrial)	Tumor cells	4	1.15(0.86,1.54)	0.344	85.6	0.000
		TICs	3	2.37(1.47,3.83)	**0.000**	0.0	0.464

FIGO, International Federation of Gynecology and Obstetrics; LNM, lymph node metastasis; LVSI, lymphovascular space invasion; RR, relative risk; CI, confidence interval; IHC, immunohistochemistry; TICs, tumor-infiltrating immune cells. P_Z_, p-value for association; P_H_, p-value for heterogeneity obtained by Q-test; I^2^, the degree of heterogeneity by I^2^ statistic. Bold indicated the significance after analysis of two or more than two studies (p < 0.05).

#### Subgroup Analysis in All and Each Cancer Type

**Figure 5:**
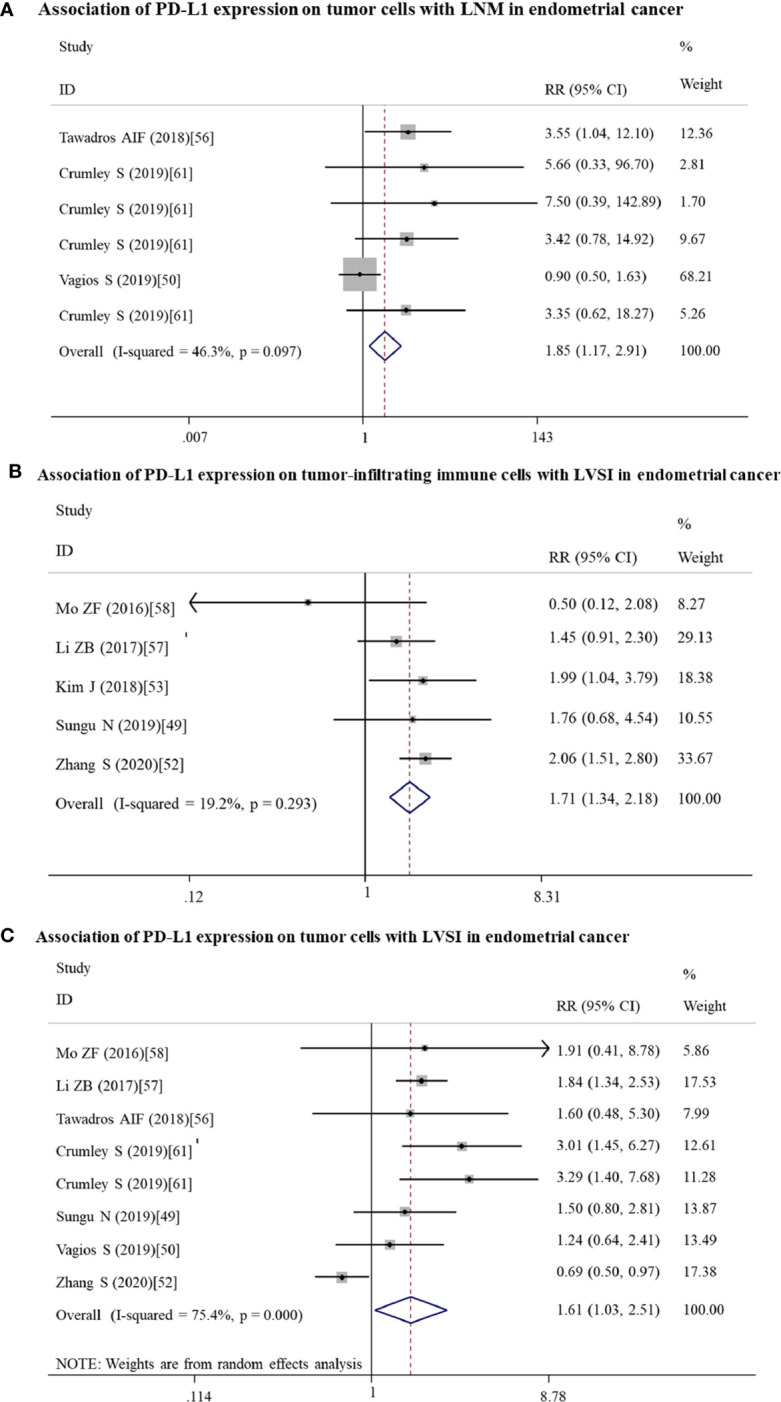
Forest plots showing the association of PD-L1 expression for endometrial cancer patients. **(A)** PD-L1 expression on tumor cells and LNM. **(B)** PD-L1 expression on tumor-infiltrating immune cells and LVSI. **(C)** PD-L1 expression on tumor cells and LVSI. LNM, lymph node metastasis. LVSI, lymphovascular space invasion; HR, hazard ratio; RR, relative risk; CI, confidence interval.

High expressed PD-L1 could predict LNM for ovarian (n = 4: RR = 1.70; 95% CI = 1.23 – 2.34, p = 0.001) and endometrial (n = 6: RR = 1.85; 95% CI = 1.17 – 2.91, p = 0.008) cancer patients. These associations for the high risk of LNM may be mainly resulted from the upregulated expression of PD-L1 on tumor cells (ovarian: n = 4, RR = 1.70; 95% CI = 1.23 – 2.34, p = 0.001; **Figure 4B**; endometrial: n = 6, RR = 1.85; 95% CI = 1.17 – 2.91, p = 0.008; **Figure 5A**).

High expressed PD-L1 also could predict high FIGO stage for ovarian (III–IV vs I–II: n = 21, RR = 1.14; 95% CI = 1.01 – 1.29, p = 0.039) and endometrial cancer (II–IV vs I: n = 4, RR = 2.90; 95% CI = 1.70 – 4.94, p = 0.000). PD-L1 may be mainly high expressed on tumor cells (III-IV vs I-II: n = 14, RR = 1.23; 95% CI = 1.12 – 1.36, p = 0.000; **Figure 4C**) in ovarian patients, while both tumor cells (II–IV vs I: n = 2, RR = 2.96; 95% CI = 1.58 – 5.55, p = 0.001) and TICs (III–IV vs I–II: n = 2, RR = 1.72; 95% CI = 1.16 – 2.54, p = 0.007; II–IV vs I: n = 2, RR = 3.47; 95% CI = 1.23 – 9.83, p = 0.019) expressed in endometrial cancer patients.

Likewise, endometrial cancer patients may have LVSI (n = 14, RR = 1.51; 95% CI = 1.15 – 2.00, p = 0.004) if PD-L1 was high expressed on TICs (n = 5: RR =1.71; 95% CI = 1.34 – 2.18, p = 0.000; **Figure 5B**) or tumor cells (n = 8: RR = 1.61; 95% CI = 1.03 – 2.51, p = 0.035; **Figure 5C**).

PD-L1 high expressed on TICs was associated with increasing infiltration depth (n = 2: RR = 1.77; 95% CI = 1.33 – 2.35, p = 0.000) and grade (n = 3: RR = 2.37; 95% CI = 1.47 – 3.83, p = 0.000) in endometrial cancer (**Table 4**). There was no significant relationship of PD-L1 with tumor size regardless of overall or subgroup analyses.

### Association of PD-L1 Expressions With Response to Anti-Programmed Cell Death-1/Programmed Cell Death-Ligand 1 Treatment

#### Overall Analysis in All Gynecological Cancers

**Figure 6:**
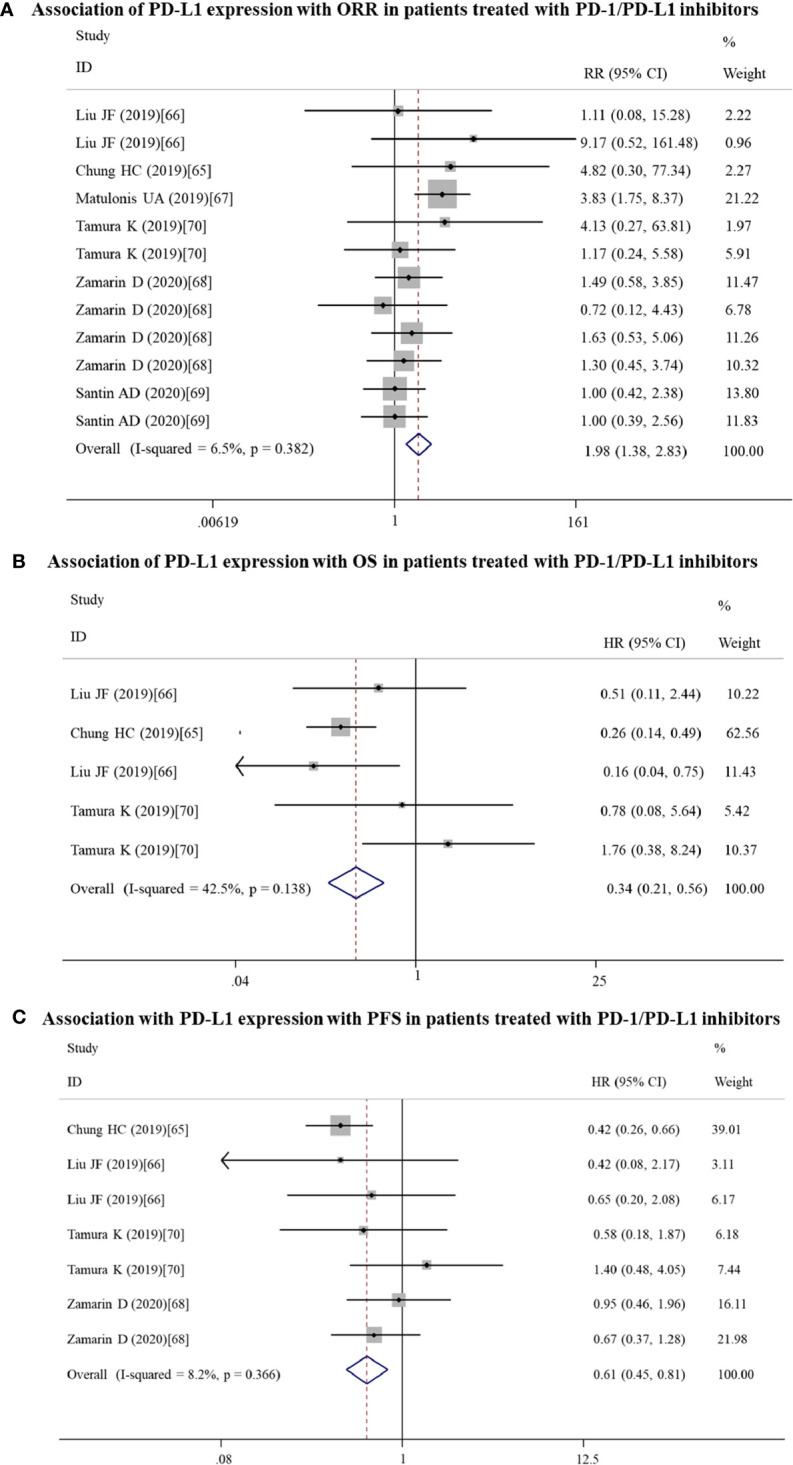
Forest plots showing the association between PD-L1 expression and response to PD-1/PD-L1 inhibitors in gynecological cancers. **(A)** Overall response rate (ORR). **(B)** Overall survival (OS). **(C)** Progression-free survival (PFS). HR, hazard ratio; CI, confidence interval.

Twelve datasets reported the ORR, while OS and PFS were recorded in 5 and 7 datasets, respectively. Meta-analysis of these datasets indicated that patients with PD-L1 positive expression may get more benefit from anti-PD-1/PD-L1 antibodies than PD-L1 negative patients, showing a higher ORR (RR = 1.98; 95% CI: 1.38 – 2.83, p = 0.000) (**Figure 6A**), longer OS (HR = 0.34; 95% CI: 0.21 – 0.56, p = 0.000) (**Figure 6B**) and PFS (HR = 0.61; 95% CI: 0.46 – 0.81, p = 0.001) (**Figure 6C**).

#### Subgroup Analysis in All Gynecological Cancers

Subgroup analysis was performed only for ORR and PFS, not OS because of small articles included. The results showed that PD-1/PD-L1 inhibitors should be especially recommended for PD-L1-positive ovarian patients who could gain the high ORR (n = 6: RR = 2.17; 95% CI: 1.38 – 3.42, p = 0.001) and PD-L1-positive cervical patients who could obtain a longer PFS (n = 2: RR = 0.44; 95% CI: 0.29 – 0.68, p = 0.000) (**Table 5**).

**Table 5:** Subgroup analysis in response to PD-1/PD-L1 inhibitors.

	Comparison		Studies	RR(95%CI)	*P_z_-*value	I^2^	*P_H_*-value
ORR	Cancer type	Ovarian	6	2.17(1.38,3.42)	**0.001**	7.9	0.366
		Cervical	2	4.50(0.63,32.01)	0.133	0.0	0.937-
		Endometrial	4	1.27(0.72,2.25)	0.410	0.0	0.498
	IHC detection area	Tumor cells	5	1.27(0.72,2.24)	0.403	0.0	0.827
		TICs	5	1.50(0.85,2.65)	0.163	0.0	0.665
		Tumor cells + TICs	2	3.92(1.84,8.38)	**0.000**	0.0	0.874
PFS	Cancer type	Ovarian	3	0.74(0.47,1.17)	0.196	0.0	0.604
		Cervical	2	0.44(0.29,0.68)	**0.000**	0.0	0.616
		Endometrial	2	0.99(0.45,2.18)	0.977	0.0	0.342
	IHC detection area	Tumor cells	3	0.95(0.55,1.61)	0.835	0.0	0.551
		TICs	3	0.64(0.38,1.07)	0.088	0.0	0.873
		Tumor cells + TICs	1	0.42(0.26,0.67)	0.000	–	–

PFS, progression free survival; ORR, overall response rate; RR, relative risk; CI, confidence interval; IHC, immunohistochemistry; TICs, tumor-infiltrating immune cells. P_Z_, p-value for association; P_H_, p-value for heterogeneity obtained by Q-test; I^2^, the degree of heterogeneity by I^2^ statistic. Bold indicated the significance after analysis of two or more than two studies (p < 0.05).

#### Publication Bias and Sensitivity Analyses

Although significant heterogeneities were present for analysis of OS, PFS, DFS, LNM, FIGO stage, infiltration depth, LVSI and grade, Egger’s linear regression test analysis showed that there were no publication bias among their related studies (OS: p = 0.478; PFS, p = 0.939; DFS, p = 0.534; LNM, p = 0.917; FIGO stage, p = 0.087; infiltration depth, p = 0.181; LVSI, p = 0.504; grade, p = 0.246), indicating the credibility of results. Sensitivity analyses also confirmed the robustness of the results.

## Discussion

There were several meta-analyses to analyze the prognostic significance PD-L1 by integrating multiple solid tumor types (71–74), but rare studies included the gynecological cancer [n = 1, cervical carcinoma (73, 75); n = 1 each for cervical and ovarian cancer (74)]. Our present study, for the first time, specifically investigated the association of PD-L1 expression with the prognosis and clinicopathological factors in all gynecological cancer patients. Pooled results showed that PD-L1 overexpression was not associated with OS, PFS, RFS, CSS and DFS, but subgroup analysis suggested PD-L1 overexpression predicted shorter OS in studies with reported HR and the cut-off value of 5%. Furthermore PD-L1 overexpression predicted clinical malignant characteristics of gynecological cancer patients (including LNM, advanced FIGO stage and LVSI). These conclusions seemed to be in line with the results of previous meta-studies of clinical samples (71–74) and the tumor-promoting mechanisms demonstrated by *in vitro* and *in vivo* experiments. For example, Wang et al. found that overexpression of PD-L1 significantly increased the migration, invasion, proliferative and colony-forming abilities of Siha and Me180 cervical cancer cell lines compared with control. Tumor xenograft growth was also significantly enhanced and LNM was more apparently observed in abdominal cavities of mice injected with PD-L1-overexpressing cervical cancer cells (16). Fei et al. also demonstrated that ectopic expression of PD-L1 promoted nasopharyngeal carcinoma cell invasion and metastasis *in vitro* and *in vivo*, which was attributed to its capability to activate the epithelial-mesenchymal transition process in a PI3K/AKT-dependent manner (76).

Although previous meta-analysis studies had investigated the prognostic and clinicopathological impact of PD-L1 for cervical (10), ovarian (12) and endometrial cancer (11), the number of articles included was relatively small. Our study performed an updated meta-analysis for each gynecological cancer type by increasing the number of articles included by more than two fold. As expected, some of our results were obviously different from previous reports: our analysis showed that PD-L1 was not significantly associated with OS and PFS in any cancer type, but the study of Gu et al. reported PD-L1 overexpression was related to a poor OS in patients with cervical cancer (10); our results revealed that LNM, high FIGO stage and LVSI were more frequently observed in PD-L1-positive endometrial cancer patients compared with negative controls; while Lu et al. proved that elevated PD-L1 expression was only correlated with advanced stage, but not LVSI (11). Thus, we consider our conclusions may be more believable by analysis of larger samples. Furthermore, compared with the above mete-analyses (10, 11), one innovation point in our study was to collect the PD-L1 expression on both of tumor cells and TICs, not only tumor cells. As anticipated, we obtained several new conclusions: high expression of PD-L1 on TICs was a protective factor for a poor OS in ovarian cancer patients (HR < 1), but a risk factor for unfavorable OS in cervical cancer patients, advanced stage, LVSI, high grade and increasing infiltration depth in endometrial cancer patients (HR > 1). Positive expression of PD-L1 on tumor cells was associated with a poor OS for ovarian cancer patients, LVSI for endometrial cancer patients, LNM and advanced stage for both cancer types. The anti-tumor roles of high PD-L1 on TICs for ovarian patients was also illustrated in other cancers, including colorectal (77), breast (78) and high-grade neuroendocrine carcinoma of lung (79). Its anti-cancer effects may be related with an adaptive mechanism to further activate and increase levels of cytotoxic CD8+ T cells as well as tumor-infiltrating lymphocytes (78, 80–82). Also, there was a study of non-small cell lung cancer to report that PD-L1 expression on tumor cells and TICs was associated with high levels of M2 tumor-associated macrophages and then led to a poor prognosis and an aggressive malignant phenotype, which may be one potential reason to cause the tumor-promoting effects of PD-L1 on tumor cells and TICs for gynecological cancers (83, 84).

In consideration of the fact that PD-L1 was highly expressed and the use of anti-PD-L1/PD-1 antibodies induced cell apoptosis and cell-cycle arrest in G0/G1 phase in gynecological cancer cells (85), increasing scholars recommended to using the PD-L1/PD-1 immune checkpoint inhibitors for the treatment of gynecological cancers in clinic (4, 86). However, like other therapeutic methods, there were differences in the therapeutic efficiency among different patients (69). Thus, it is also necessary to explore biomarkers to distinguish the patients and then schedule the PD-L1/PD-1 immune checkpoint inhibitors more reasonably. Previous studies on other cancers suggested the magnitude of clinical benefit from PD-L1/PD-1 inhibitors was PD-L1-dependent (87, 88). Therefore, we also investigated the associations between PD-L1 expression and ORR, OS, PFS in gynecological cancer patients. In agreement with the above studies (87–89), we also found PD-L1 patients had a significantly higher ORR (especially ovarian cancer), OS and PFS (especially cervical cancer) than PD-L1-negative patients. Although Kowanetz et al. observed that the ORR was relatively lower in patients with tumors expressing high PD-L1 levels on tumor cells than TICs (40% vs 22%) (80), our subgroup results indicated no association with tumor cells or TICs, which may be related with the small sample size.

Several limitations should be acknowledged in this study. First is the retrospective nature in most of included studies. Second, the cut-off value of PD-L1 was determined by different methods in included studies, which influenced its clinical use. Third, the number of included studies to report the association of PD-L1 expression with RFS/CSS/DFS/response to anti-PD-L1/PD-1 treatment was relatively small, which may compromise the credibility of the results and influence the subgroup analysis for each cancer type. Fourth, the estimation of HR from Kaplan–Meier curve may introduce some errors. Fifth, the restriction of articles published in other languages may lead to some negative results neglected.

## Conclusion

**Figure 7:**
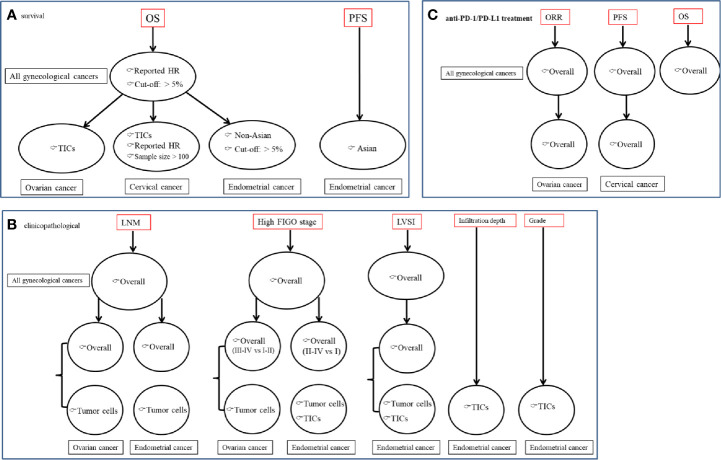
A summary figure to show the crucial results to demonstrate the predictive values of PD-L1 for gynecological cancers patients. **(A)** associated with survival; **(B)** associated with clinicopathological features; **(C)** associated with anti-PD-1/PD-L1 treatment effects.

Our meta-analyses (**Figure 7**) indicated that positive PD-L1 detected by IHC may serve as a valuable predictor of a poor prognosis (OS, PFS), malignant clinicopathological characteristics (LNM, advanced FIGO stage and LVSI) and response efficiency to anti-PD-1/PD-L1 (ORR, OS, PFS) for patients with gynecological cancers, especially expression on tumor cells. High expressed PD-L1 on TICs may exert dual functions, including anti-cancer for ovarian cancer or oncogenic for cervical and endometrial cancers.

## Data Availability Statement

The original contributions presented in the study are included in the article/**Supplementary Material**; further inquiries can be directed to the corresponding author.

## Author Contributions

CZ and QY conceived and designed the study, collected the data, and performed the analysis. CZ wrote the first draft of the manuscript. QY was involved in the interpretation of the analyses and revised the manuscript. All authors contributed to the article and approved the submitted version.

## Conflict of Interest

The authors declare that the research was conducted in the absence of any commercial or financial relationships that could be construed as a potential conflict of interest.

## Supplementary Material

Supplementary Table 1 The data extracted from the published studies.

Supplementary Table 2 The Newcastle-Ottawa scale (NOS) quality assessment of the enrolled studies.

Supplementary Table 3 Subgroup analysis on the outcome of OS in each cancer type.

Supplementary Table 4 Subgroup analysis on the outcome of PFS in each cancer type.

## Abbreviations

PD-L1, programmed death-ligand 1; PD-1, programmed cell death-1; IHC, immunohistochemistry; TICs, tumor-infiltrating immune cells; OS, overall survival; HR, hazard ratios; CI, confidence interval; PFS, progression free survival; PRISMA, Preferred Reporting Items for Systematic Review and Meta-analysis; RFS, recurrence-free survival; CSS, cancer-specific survival; DFS, disease-free survival; FIGO, International Federation of Gynecology and Obstetrics; LNM, lymph node metastasis; LVSI, lymphovascular space invasion; ORR, overall response rate; NOS, Newcastle-Ottawa Scale; RR, relative risks.
