# Supplementary material for: Predictive Values of Programmed Cell Death-Ligand 1 Expression for Prognosis, Clinicopathological Factors, and Response to Programmed Cell Death-1/Programmed Cell Death-Ligand 1 Inhibitors in Patients With Gynecological Cancers: A Meta-Analysis
Source: Front Oncol. 2021 Feb 1;10:572203. doi: 10.3389/fonc.2020.572203 (PMC7901918; doi:10.3389/fonc.2020.572203)
Supplement: Supplementary Table 2 — The Newcastle-Ottawa scale (NOS) quality assessment of the enrolled studies. [file Table_2.docx]

**Table S2 The Newcastle-Ottawa scale (NOS) quality assessment of the enrolled studies**

| Study | Selection (score) | | | |  | Comparability |  | Exposure (score) | | |  | Total score |
| --- | --- | --- | --- | --- | --- | --- | --- | --- | --- | --- | --- | --- |
|  | Representativeness of the exposed cohort | Selection of the non-exposed cohort | Ascertainment of exposure | Demonstration that  outcome of interest was not present  at start of study |  | Comparability of cohorts on the basis of the design or analysis |  | Assessment of outcome | Follow-up long enough for outcomes to occur | Adequacy of follow up of cohorts |  |  |
| Wang S | 1 | 1 | 1 | 1 |  | 1 |  | 1 | 1 | 1 |  | 8 |
| Enwere EK | 1 | 1 | 1 | 1 |  | 2 |  | 1 | 1 | 1 |  | 9 |
| Feng M | 1 | 1 | 1 | 1 |  | 2 |  | 1 | 1 | 1 |  | 9 |
| Kim M | 1 | 1 | 1 | 1 |  | 2 |  | 1 | 1 | 1 |  | 9 |
| Iijima M | 1 | 1 | 1 | 1 |  | 2 |  | 1 | 1 | 1 |  | 9 |
| Tsuchiya T | 1 | 1 | 1 | 1 |  | 2 |  | 1 | 1 | 1 |  | 9 |
| Kawachi A1 | 1 | 1 | 1 | 1 |  | 2 |  | 1 | 1 | 1 |  | 9 |
| Loharamtaweethong K | 1 | 1 | 1 | 1 |  | 1 |  | 1 | 1 | 1 |  | 8 |
| Miyasaka Y | 1 | 1 | 1 | 1 |  | 1 |  | 1 | 1 | 1 |  | 8 |
| Chen H | 1 | 1 | 1 | 1 |  | 1 |  | 1 | 1 | 1 |  | 8 |
| Lippens L | 1 | 1 | 1 | 1 |  | 1 |  | 1 | 1 | 1 |  | 8 |
| Karim R | 1 | 1 | 1 | 1 |  | 2 |  | 1 | 1 | 1 |  | 9 |
| Loharamtaweethong K | 1 | 1 | 1 | 1 |  | 1 |  | 1 | 1 | 1 |  | 8 |
| Grochot RM | 1 | 1 | 1 | 1 |  | 1 |  | 1 | 1 | 0 |  | 7 |
| Xu M | 1 | 1 | 1 | 1 |  | 2 |  | 1 | 1 | 1 |  | 9 |
| Nhokaew W | 1 | 1 | 1 | 1 |  | 2 |  | 1 | 1 | 0 |  | 8 |
| Schmoeckel E | 1 | 1 | 1 | 1 |  | 1 |  | 1 | 1 | 1 |  | 8 |
| Hamanishi J | 1 | 1 | 1 | 1 |  | 2 |  | 1 | 1 | 1 |  | 9 |
| Mesnage SJL | 1 | 1 | 1 | 1 |  | 1 |  | 1 | 1 | 1 |  | 8 |
| Zhu J | 1 | 1 | 1 | 1 |  | 1 |  | 1 | 1 | 1 |  | 8 |
| Zhu J | 1 | 1 | 1 | 1 |  | 2 |  | 1 | 1 | 0 |  | 8 |
| Zong L | 1 | 1 | 1 | 1 |  | 1 |  | 1 | 1 | 1 |  | 8 |
| Wang Q | 1 | 1 | 1 | 1 |  | 2 |  | 1 | 1 | 1 |  | 9 |
| Zhu X | 1 | 1 | 1 | 1 |  | 2 |  | 1 | 1 | 1 |  | 9 |
| Buderath P | 1 | 1 | 1 | 1 |  | 1 |  | 1 | 1 | 1 |  | 8 |
| Kim KH | 1 | 1 | 1 | 1 |  | 1 |  | 1 | 1 | 1 |  | 8 |
| Zhu X | 1 | 1 | 1 | 1 |  | 2 |  | 1 | 1 | 1 |  | 9 |
| Zhang L | 1 | 1 | 1 | 1 |  | 2 |  | 1 | 1 | 1 |  | 9 |
| Alldredge J | 1 | 1 | 1 | 1 |  | 2 |  | 1 | 1 | 1 |  | 9 |
| De La Motte Rouge T | 1 | 1 | 1 | 1 |  | 1 |  | 1 | 1 | 1 |  | 8 |
| Martin de la Fuente L | 1 | 1 | 1 | 1 |  | 2 |  | 1 | 1 | 1 |  | 9 |
| Chatterjee J | 1 | 1 | 1 | 1 |  | 1 |  | 1 | 1 | 0 |  | 7 |
| Henriksen JR | 1 | 1 | 1 | 1 |  | 2 |  | 1 | 1 | 1 |  | 9 |
| Sungu N | 1 | 1 | 1 | 1 |  | 1 |  | 1 | 1 | 1 |  | 8 |
| Vagios S | 1 | 1 | 1 | 1 |  | 1 |  | 1 | 1 | 1 |  | 8 |
| Kucukgoz Gulec U | 1 | 1 | 1 | 1 |  | 1 |  | 1 | 1 | 1 |  | 8 |
| Zhang S | 1 | 1 | 1 | 1 |  | 2 |  | 1 | 1 | 1 |  | 9 |
| Kim J | 1 | 1 | 1 | 1 |  | 2 |  | 1 | 1 | 1 |  | 9 |
| Jones TE | 1 | 1 | 1 | 1 |  | 1 |  | 1 | 1 | 1 |  | 8 |
| Kucukgoz Gulec U | 1 | 1 | 1 | 1 |  | 1 |  | 1 | 1 | 1 |  | 8 |
| Chung HC | 1 | 1 | 1 | 1 |  | 1 |  | 1 | 1 | 0 |  | 7 |
| Liu JF | 1 | 1 | 1 | 1 |  | 1 |  | 1 | 1 | 0 |  | 7 |
| Matulonis UA | 1 | 1 | 1 | 1 |  | 1 |  | 1 | 1 | 0 |  | 7 |
| Zamarin D | 1 | 1 | 1 | 1 |  | 1 |  | 1 | 1 | 1 |  | 8 |
| Tawadros AI | 1 | 1 | 1 | 1 |  | 2 |  | 1 | 1 | 0 |  | 8 |
| Li ZB | 1 | 1 | 1 | 1 |  | 1 |  | 1 | 1 | 0 |  | 7 |
| Mo ZF | 1 | 1 | 1 | 1 |  | 2 |  | 1 | 1 | 0 |  | 8 |
| Yamashita H | 1 | 1 | 1 | 1 |  | 1 |  | 1 | 1 | 1 |  | 8 |
| Engerud H | 1 | 1 | 1 | 1 |  | 2 |  | 1 | 1 | 1 |  | 9 |
| Crumley S | 1 | 1 | 1 | 1 |  | 1 |  | 1 | 1 | 1 |  | 8 |
| Santin AD | 1 | 1 | 1 | 1 |  | 1 |  | 1 | 1 | 1 |  | 8 |
| Li MJ | 1 | 1 | 1 | 1 |  | 1 |  | 1 | 1 | 1 |  | 8 |
| Webb | 1 | 1 | 1 | 1 |  | 1 |  | 1 | 1 | 0 |  | 7 |
| Xue CY | 1 | 1 | 1 | 1 |  | 2 |  | 1 | 1 | 1 |  | 9 |
| Tamura K | 1 | 1 | 1 | 1 |  | 1 |  | 1 | 1 | 0 |  | 7 |
