# Supplementary material for: Predictive Values of Programmed Cell Death-Ligand 1 Expression for Prognosis, Clinicopathological Factors, and Response to Programmed Cell Death-1/Programmed Cell Death-Ligand 1 Inhibitors in Patients With Gynecological Cancers: A Meta-Analysis
Source: Front Oncol. 2021 Feb 1;10:572203. doi: 10.3389/fonc.2020.572203 (PMC7901918; doi:10.3389/fonc.2020.572203)
Supplement: Supplementary Table 3 — Subgroup analysis on the outcome of OS in each cancer type. [file Table_3.docx]

**Table S3 Subgroup analysis on the outcome of OS in each cancer type**

| Cancer | Comparison |  | Studies | HR(95%CI) | *P_z_*-value | I^2^ | *P_H_*-value |
| --- | --- | --- | --- | --- | --- | --- | --- |
| Ovarian | Region | Asian | 14 | 1.12(0.81,1.56) | 0.480 | 66.1 | 0.000 |
|  |  | Non-Asian | 8 | 0.87(0.58,1.30) | 0.496 | 77.4 | 0.000 |
|  | Sample size | <100 | 8 | 1.14(0.64,2.01) | 0.669 | 75.4 | 0.000 |
|  |  | >100 | 14 | 0.97(0.74,1.27) | 0.835 | 67.4 | 0.000 |
|  | IHC counting method | SI | 4 | 0.84(0.24,2.97) | 0.789 | 78.6 | 0.003 |
|  |  | SP | 14 | 1.00(0.75,1.34) | 0.996 | 71.9 | 0.000 |
|  |  | IRS | 3 | 1.30(0.87,1.93) | 0.198 | 50.5 | 0.133 |
|  |  | Other | 1 | 0.37(0.09,1.56) | 0.176 | - | - |
|  | Cut-off values | 1% | 4 | 0.97(0.52,1.83) | 0.932 | 85.5 | 0.000 |
|  |  | 5% | 2 | 1.19(0.80,1.78) | 0.394 | 0.0 | 0.625 |
|  |  | Others | 16 | 1.01(0.73,1.38) | 0.968 | 68.8 | 0.000 |
|  | Antibody type | Monoclonal | 20 | 0.98(0.76,1.27) | 0.888 | 70.5 | 0.000 |
|  |  | Unclear | 2 | 1.41(0.60,3.33) | 0.436 | 75.2 | 0.044 |
|  | Antibody source | Mouse | 3 | 0.97(0.73,1.29) | 0.820 | 77.9 | 0.011 |
|  |  | Rabbit | 17 | 0.99(0.78,1.26) | 0.844 | 70.8 | 0.000 |
|  |  | Unclear | 2 | 1.41(0.60,3.33) | 0.436 | 75.2 | 0.044 |
|  | IHC detection area | Tumor cells | 12 | 1.40(0.98,1.98) | 0.064 | 68.7 | 0.000 |
|  |  | TICs | 8 | 0.72(0.59,0.87) | **0.001** | 42.0 | 0.098 |
|  |  | Tumor cells + TICs | 2 | 1.07(0.66,1.73) | 0.790 | 42.5 | 0.187 |
|  | HR method | MV | 11 | 1.17(0.78,1.77) | 0.454 | 79.6 | 0.000 |
|  |  | UV | 11 | 0.90(0.68,1.19) | 0.458 | 49.1 | 0.033 |
|  | HR source | Reported | 16 | 1.11(0.83,1.47) | 0.479 | 71.2 | 0.000 |
|  |  | Estimated | 8 | 0.80(0.51,1.26) | 0.330 | 59.2 | 0.031 |
| Cervical | Region | Asian | 12 | 1.44(0.63,3.28) | 0.384 | 85.4 | 0.000 |
|  |  | Non-Asian | 4 | 0.98(0.70,1.37) | 0.911 | 0.0 | 0.794 |
|  | Sample size | <100 | 7 | 0.69(0.32,1.50) | 0.344 | 65.4 | 0.008 |
|  |  | >100 | 9 | 1.92(1.07,3.45) | **0.030** | 76.4 | 0.000 |
|  | IHC counting method | SI | 4 | 0.98(0.36,2.64) | 0.963 | 86.5 | 0.000 |
|  |  | SP | 11 | 1.32(0.68,2.59) | 0.414 | 77.1 | 0.000 |
|  |  | IRS | 1 | 6.07(1.36,26.97) | 0.018 | - | - |
|  | Cut-off values | 1% | 5 | 0.79(0.32,1.95) | 0.601 | 30.0 | 0.221 |
|  |  | 5% | 3 | 2.77(0.72,10.66) | 0.138 | 80.9 | 0.005 |
|  |  | Others | 8 | 1.15(0.64,2.07) | 0.638 | 76.9 | 0.000 |
|  | Antibody type | Monoclonal | 15 | 1.20(0.69,2.10) | 0.523 | 81.5 | 0.000 |
|  |  | Unclear | 1 | 6.07(1.36,26.97) | 0.018 | - | - |
|  | Antibody source | Mouse | 2 | 0.59(0.10,3.48) | 0.560 | 84.1 | 0.012 |
|  |  | Rabbit | 13 | 1.38(0.80,2.36) | 0.248 | 75.6 | 0.000 |
|  |  | Unclear | 1 | 6.07(1.36,26.97) | 0.018 | - | - |
|  | IHC detection area | Tumor cells | 12 | 1.23(0.70,2.15) | 0.477 | 74.7 | 0.000 |
|  |  | TICs | 3 | 3.43(1.60,7.34) | **0.000** | 23.8 | 0.269 |
|  |  | Tumor cells + TICs | 1 | 0.26(0.14,0.49) | 0.000 | - | - |
|  | HR method | MV | 3 | 2.54(0.54,11.89) | 0.238 | 62.7 | 0.069 |
|  |  | UV | 13 | 1.16(0.65,2.08) | 0.621 | 82.7 | 0.000 |
|  | HR source | Reported | 8 | 1.89(1.06,3.36) | **0.031** | 63.1 | 0.008 |
|  |  | Estimated | 8 | 0.86(0.32,2.30) | 0.760 | 88.2 | 0.000 |
| Endometrial | Region | Asian | 6 | 0.70(0.44,1.13) | 0.143 | 33.4 | 0.186 |
|  |  | Non-Asian | 7 | 1.60(1.07,2.40) | **0.022** | 38.4 | 0.136 |
|  | Sample size | <100 | 5 | 1.95(1.18,3.23) | **0.009** | 34.9 | 0.189 |
|  |  | >100 | 8 | 0.82(0.55,1.20) | 0.303 | 33.9 | 0.000 |
|  | IHC counting method | SI | 4 | 1.11(0.40,3.07) | 0.837 | 13.2 | 0.326 |
|  |  | SP | 7 | 1.75(1.17,2.62) | **0.007** | 19.9 | 0.277 |
|  |  | IRS | 2 | 0.53(0.31,0.91) | **0.021** | 0.5 | 0.316 |
|  | Cut-off values | 1% | 4 | 1.27(0.71,2.26) | 0.424 | 0.0 | 0.495 |
|  |  | 5% | 3 | 2.37(1.35,4.18) | **0.003** | 28.4 | 0.248 |
|  |  | Others | 6 | 0.62(0.39,1.00) | 0.051 | 17.5 | 0.301 |
|  | Antibody type | Monoclonal | 13 | 1.23(0.77,1.98) | 0.381 | 50.0 | 0.020 |
|  | Antibody source | Mouse | 3 | 2.84(1.49,5.41) | **0.001** | 0.0 | 0.484 |
|  |  | Rabbit | 10 | 0.86(0.61,1.22) | 0.403 | 27.2 | 0.194 |
|  | IHC detection area | Tumor cells | 7 | 1.09(0.72,1.64) | 0.698 | 68.3 | 0.004 |
|  |  | TICs | 5 | 1.16(0.68,1.99) | 0.584 | 19.2 | 0.292 |
|  |  | Tumor cells + TICs | 1 | 1.30(0.52,3.24) | 0.581 | - | - |
|  | HR method | MV | 7 | 1.13(0.76,1.68) | 0.557 | 70.5 | 0.002 |
|  |  | UV | 6 | 1.14(0.70,1.84) | 0.597 | 0.0 | 0.598 |
|  | HR source | Reported | 9 | 1.14(0.80,1.64) | 0.471 | 61.0 | 0.009 |
|  |  | Estimated | 4 | 1.11(0.62,1.98) | 0.738 | 14.4 | 0.320 |

OS, overall survival; UV, univariate analysis; MV, multivariate analysis; SP, staining percentage; SI, staining intensity score; IRS, immunoreactive SI (that is, IRS = SI × SP); HR, hazard ratios; CI, confidence interval; IHC, immunohistochemistry; TICs, tumor-infiltrating immune cells. P_Z_, p-value for association; P_H_, p-value for heterogeneity obtained by Q-test; I^2^, the degree of heterogeneity by I^2^ statistic. Bold indicated the significance after analysis of two or more than two studies (p < 0.05).
