# Supplementary material for: Predictive Values of Programmed Cell Death-Ligand 1 Expression for Prognosis, Clinicopathological Factors, and Response to Programmed Cell Death-1/Programmed Cell Death-Ligand 1 Inhibitors in Patients With Gynecological Cancers: A Meta-Analysis
Source: Front Oncol. 2021 Feb 1;10:572203. doi: 10.3389/fonc.2020.572203 (PMC7901918; doi:10.3389/fonc.2020.572203)
Supplement: Supplementary Table 4 — Subgroup analysis on the outcome of PFS in each cancer type. [file Table_4.docx]

**Table S4 Subgroup analysis on the outcome of PFS in each cancer type**

| Cancer | Comparison |  | Studies | HR(95%CI) | *P_z_*-value | I^2^ | *P_H_*-value |
| --- | --- | --- | --- | --- | --- | --- | --- |
| Ovarian | Region | Asian | 5 | 1.52(0.90,2.56) | 0.115 | 74.0 | 0.004 |
|  |  | Non-Asian | 5 | 1.04(1.00,1.07) | **0.032** | 0.0 | 0.466 |
|  | Sample size | <100 | 7 | 1.16(0.78,1.71) | 0.461 | 67.9 | 0.005 |
|  |  | >100 | 3 | 1.09(0.66,1.81) | 0.732 | 60.1 | 0.082 |
|  | IHC counting method | SI | 3 | 1.21(0.58,2.54) | 0.606 | 64.5 | 0.060 |
|  |  | SP | 5 | 0.98(0.67,1.42) | 0.906 | 38.1 | 0.168 |
|  |  | IRS | 2 | 1.47(0.47,4.57) | 0.504 | 89.4 | 0.002 |
|  | Cut-off values | 1% | 2 | 0.91(0.59,1.40) | 0.651 | 0.0 | 0.868 |
|  |  | 5% | 1 | 0.78(0.34,1.81) | 0.562 | - | - |
|  |  | Others | 7 | 1.04(1.01,1.08) | **0.012** | 73.7 | 0.001 |
|  | Antibody type | Monoclonal | 7 | 1.06(0.71,1.59) | 0.771 | 51.0 | 0.057 |
|  |  | Unclear | 3 | 1.26(0.76,2.10) | 0.376 | 82.5 | 0.003 |
|  | Antibody source | Mouse | 1 | 2.57(1.11,5.94) | 0.027 | - | - |
|  |  | Rabbit | 6 | 0.94(0.66,1.35) | 0.746 | 32.7 | 0.190 |
|  |  | Unclear | 3 | 1.26(0.76,2.10) | 0.376 | 82.5 | 0.003 |
|  | IHC detection area | Tumor cells | 5 | 1.57(0.93,2.64) | 0.089 | 70.4 | 0.009 |
|  |  | TICs | 4 | 0.96(0.77,1.20) | 0.697 | 14.9 | 0.318 |
|  |  | Tumor cells + TICs | 1 | 0.88(0.51,1.51) | 0.644 | - | - |
|  | HR method | MV | 3 | 1.53(0.75,3.12) | 0.244 | 73.3 | 0.024 |
|  |  | UV | 7 | 1.03(0.75,1.41) | 0.867 | 58.8 | 0.024 |
|  | HR source | Reported | 8 | 1.21(0.89,1.37) | 0.227 | 68.4 | 0.002 |
|  |  | Estimated | 2 | 0.82(0.49,1.51) | 0.447 | 0.0 | 0.404 |
| Cervical | Region | Asian | 6 | 0.84(0.37,1.92) | 0.673 | 76.1 | 0.001 |
|  |  | Non-Asian | 3 | 1.00(0.72,1.38) | 0.992 | 0.0 | 0.994 |
|  | Sample size | <100 | 7 | 0.85(0.43,1.69) | 0.640 | 73.1 | 0.001 |
|  |  | >100 | 2 | 1.00(0.70,1.44) | 0.999 | 0.0 | 0.914 |
|  | IHC counting method | SI | 1 | 0.42(0.26,0.67) | 0.000 | - | - |
|  |  | SP | 7 | 0.85(0.64,1.13) | 0.266 | 0.0 | 0.638 |
|  |  | IRS | 1 | 6.30(2.11,18.80) | 0.001 | - | - |
|  | Cut-off values | 1% | 4 | 0.54(0.31,0.94) | **0.029** | 0.0 | 0.859 |
|  |  | Others | 5 | 0.85(0.65,1.10) | 0.208 | 82.3 | 0.000 |
|  | Antibody type | Monoclonal | 8 | 0.71(0.55,0.90) | **0.005** | 35.1 | 0.148 |
|  |  | Unclear | 1 | 6.30(2.11,18.80) | 0.001 | - | - |
|  | Antibody source | Mouse | 1 | 0.42(0.26,0.67) | 0.000 | - | - |
|  |  | Rabbit | 7 | 0.85(0.64,1.13) | 0.266 | 0.0 | 0.638 |
|  |  | Unclear | 1 | 6.30(2.11,18.80) | 0.001 | - | - |
|  | IHC detection area | Tumor cells | 8 | 0.97(0.74,1.27) | 0.800 | 57.2 | 0.022 |
|  |  | Tumor cells + TICs | 1 | 0.42(0.26,0.67) | 0.000 | - | - |
|  | HR method | MV | 1 | 0.62(0.16,2.43) | 0.492 | - | - |
|  |  | UV | 8 | 0.90(0.54,1.49) | 0.674 | 69.0 | 0.001 |
|  | HR source | Reported | 4 | 1.34(0.66,2.75) | 0.420 | 71.9 | 0.014 |
|  |  | Estimated | 5 | 0.54(0.38,0.76) | **0.001** | 5.7 | 0.374 |
| Endometrial | Region | Asian | 5 | 1.59(1.01,2.51) | **0.045** | 47.6 | 0.106 |
|  |  | Non-Asian | 2 | 0.55(0.27,1.14) | 0.107 | 0.0 | 0.729 |
|  | Sample size | <100 | 2 | 0.99(0.45,2.18) | 0.977 | 0.0 | 0.342 |
|  |  | >100 | 5 | 1.44(0.64,3.23) | 0.374 | 68.0 | 0.014 |
|  | IHC counting method | SI | 4 | 1.97(1.11,3.48) | **0.020** | 48.6 | 0.120 |
|  |  | SP | 3 | 0.76(0.45,1.29) | 0.132 | 3.1 | 0.356 |
|  | Cut-off values | 1% | 2 | 0.77(0.39,1.55) | 0.471 | 51.5 | 0.151 |
|  |  | 5% | 1 | 0.75(0.34,1.66) | 0.478 | - | - |
|  |  | Others | 4 | 1.97(1.12,3.48) | **0.020** | 48.6 | 0.120 |
|  | Antibody type | Monoclonal | 7 | 1.27(0.70,2.30) | 0.431 | 56.1 | 0.034 |
|  | Antibody source | Mouse | 1 | 0.50(0.20,1.25) | 0.138 | - | - |
|  |  | Rabbit | 6 | 1.42(0.93,2.17) | 0.108 | 47.8 | 0.088 |
|  | IHC detection area | Tumor cells | 4 | 0.93(0.54,1.61) | 0.795 | 27.6 | 0.246 |
|  |  | TICs | 3 | 2.09(0.62,6.99) | 0.233 | 65.3 | 0.056 |
|  | HR method | MV | 3 | 1.85(0.42,8.08) | 0.415 | 80.4 | 0.006 |
|  |  | UV | 4 | 1.02(0.63,1.66) | 0.938 | 0.0 | 0.455 |
|  | HR source | Reported | 4 | 1.78(0.65,4.89) | 0.263 | 70.8 | 0.016 |
|  |  | Estimated | 3 | 0.86(0.49,1.71) | 0.603 | 0.0 | 0.567 |

OS, overall survival; UV, univariate analysis; MV, multivariate analysis; SP, staining percentage; SI, staining intensity score; IRS, immunoreactive SI (that is, IRS = SI × SP); HR, hazard ratios; CI, confidence interval; IHC, immunohistochemistry; TICs, tumor-infiltrating immune cells. P*_Z_*, p-value for association; P*_H_*, p-value for heterogeneity obtained by Q-test; I^2^, the degree of heterogeneity by I2 statistic. Bold indicated the significance after analysis of two or more than two studies (p < 0.05).
